# Survival rate in acute kidney injury superimposed COVID-19 patients: a systematic review and meta-analysis

**DOI:** 10.1080/0886022X.2020.1756323

**Published:** 2020-04-27

**Authors:** Hatem Ali, Ahmed Daoud, Mahmoud M. Mohamed, Sohail Abdul Salim, Lenar Yessayan, Jyoti Baharani, Asam Murtaza, Vinaya Rao, Karim M. Soliman

**Affiliations:** a Department of Medicine, Division of Nephrology, University Hospitals of Birmingham, UK; b Department of Medicine, Division of Nephrology, Faculty of Medicine, Cairo University, Egypt; c Department of Medicine, Division of Nephrology, University of Tennessee, USA; d Department of Medicine, Division of Nephrology, University of Mississippi Medical Center, Jackson, MS, USA; e Department of Medicine, Division of Nephrology, University of Michigan, Ann Arbor, MI, USA; f Department of Medicine, Division of Nephrology, Walsall Manor Hospital, UK; g Department of Medicine, Division of Nephrology, Medical University of South Carolina, USA

**Figure 1.**
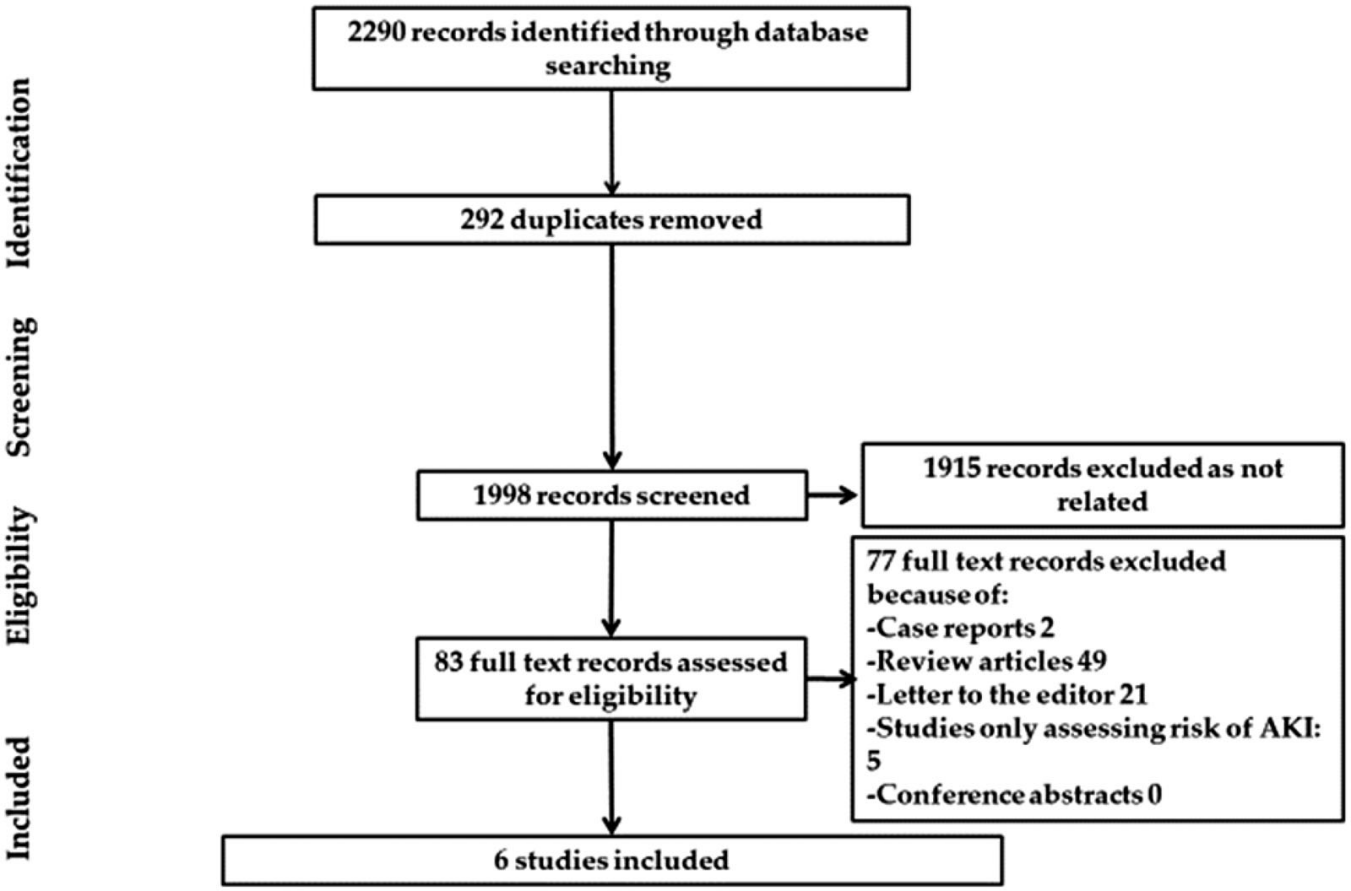
PRISMA diagram for the systematic review.

**Figure 2.**
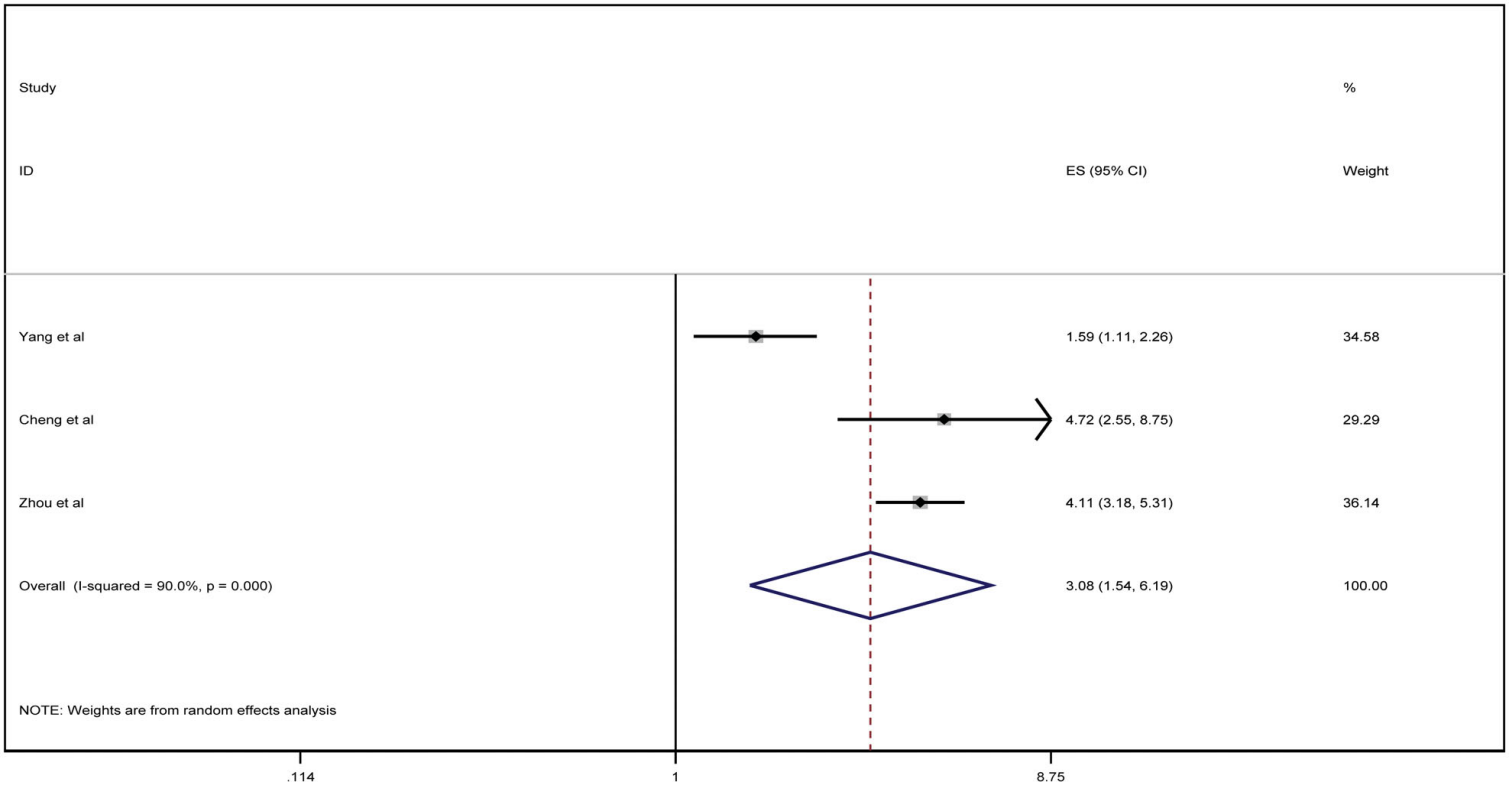
Forest plot analysis.

**Figure 3.**
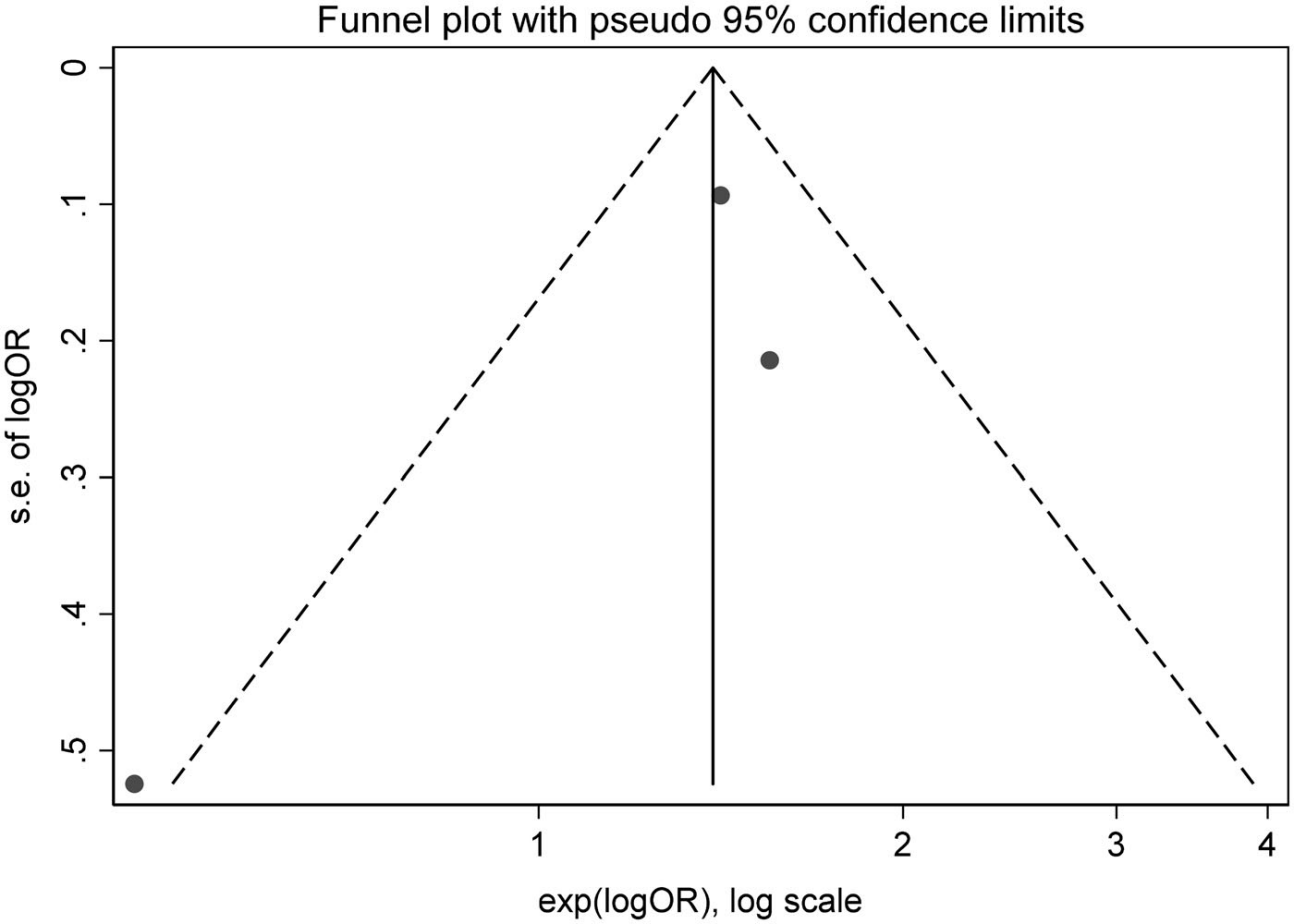
Funnel plot.

Dear Editor,

Of no doubt, the whole world is passing through a potentially life-threatening and economically destructive global pandemic caused by the novel coronavirus (COVID-19; SARS-CoV-2; previously known as 2019-nCoV) [1]. The clinical course of infection is widely unpredicted and variable, ranging from asymptomatic infection to multi-organ system failure and death [2–4]. Nevertheless, the survival rate among patients with COVID-19 and superimposed acute kidney injury (AKI) remains unclear [5,6]. Hence; we ushered a systemic review and meta-analysis exploring the survival outcome of COVID-19 subjects who developed severe AKI, the latter defined as subjects who require acute renal replacement therapy (RRT) or meet the Kidney Disease Improving Global Outcome (KDIGO) definition of AKI stage III. We included all studies performed on human beings for which baseline creatinine, occurrence of AKI stage III and/or need for acute RRT were reported and excluded case reports, review articles, or studies assessing clinical characteristics and conference abstracts. Ethical approval was not required for this work due to use of anonymous data that is publicly available. A systematic review in Pubmed, Medline, Embase and Cochrane databases to select studies that met the inclusion criteria was performed by 3 authors (H.A, M.M, A.A). The search terms used were (coronavirus, COVID-19, SARS-COV-2 and (mortality, survival, outcomes, dialysis, acute renal failure, acute kidney injury, renal replacement therapy). These search terms were individually used and then combined in different databases. References within the chosen studies were reviewed. All the included studies were reviewed by supervising authors. Any disagreement among authors collecting the data was investigated by supervising authors. Consensus among all authors was essential to include the studies in the systematic review. The following data were collected: name of the first author, journal title, publication date, place of the study, sample size, baseline creatinine, relative risk and confidence intervals for association of acute renal failure and mortality. We followed the recommendations of Cochrane collaboration and the Quality of Reporting of Meta-analyses guidelines [7,8]. STATA package-15 was used for statistical analysis. We combined all study-specific estimates using inverse-variant weighted averages of logarithmic relative risk in random effects model (REM). Confidence interval including the value of one was used evident for statistically significant estimate. Heterogeneity was evaluated using Higgins I-squared statistic. Heterogeneity was estimated when the level of *p* value was <.1. Results of the REM were spread out on the forest plot graph. The Newcastle-Ottawa score was used to evaluate the quality of the papers included. Egger's test was used to assess publication bias. A total of 2290 abstracts were reviewed. Out of six studies included in the systematic review, only three studies met the inclusion criteria and were pooled into a meta-analysis (PRISMA diagram, Figure 1). Due to lack of a controlled survival group (only severe AKI subjects were included), the studies by Zhang et al. and Shi et al. were not included in the meta-analysis [9,10]. As compared to Ruan et al., Cheng et al.’s study was more recent, included a larger sample size and since both shared the same cohort, only the latter was included in our meta-analysis [5,11]. The baseline characteristics of the studies included are shown in Table 1. The Newcastle-Ottawa score of the included studies is shown in Table 2. REM showed that severe AKI is associated with higher risk of mortality (relative risk = 3.08, confidence interval ranges from 1.54 to 6.19) as shown in Figure 2. There was evidence of heterogeneity with I-squared =90% and *p* < .001. Publication bias was shown in the funnel plot analysis in Figure 3. By applying Egger's test for assessment of bias, there was evidence of small studies effect with *p* = .93.

**Figure 4.**
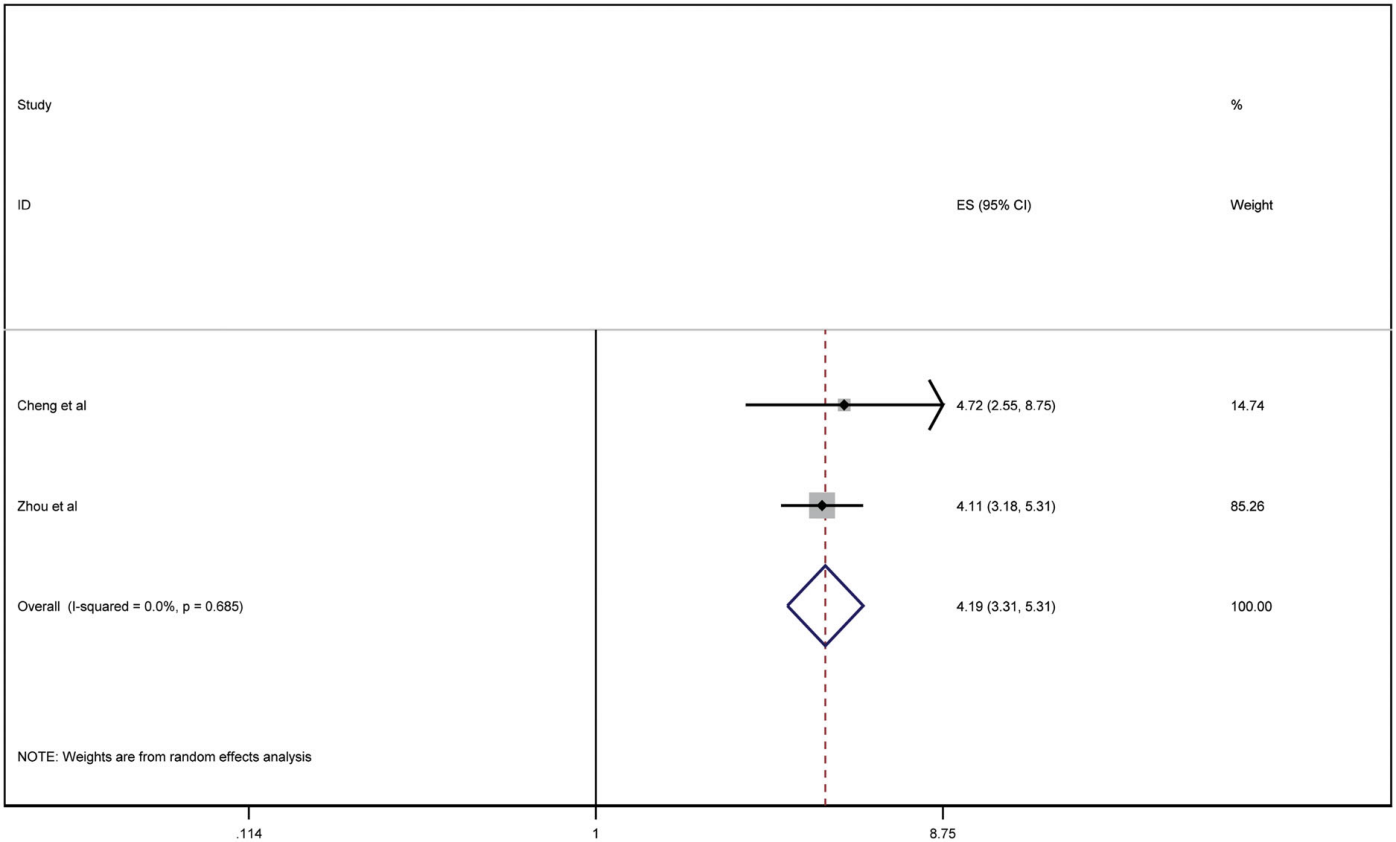
Random effects model after excluding Yang et al.

**Table 1.** Baseline characteristics of studies included in the systematic review.

Name	Journal	Date of Publication	Name of hospital	Study population	Baseline creatinine Mean (standard de*via*tion)
Yang et al.	Lancet Respiratory Medicine	March 2020	Intensive care unit (ICU) of Wuhan Jin Yin-tan hospital (Wuhan, China)	52 patients	76・3 (27・4) umol/L in survivors80・7 (32・3) umol/L in non-survivors
Cheng et al.	Kidney International	March 2020	Tongji Hospital, Wuhan, China	701 patients	77 ( 31) umol/L
Zhou et al.	Lancet	March 2020	Jinyintan Hospital and Wuhan Pulmonary Hospital-China	191 patients	8 patients had baseline creatinine >133umol/L
Zhang et al.	medRxiv	March 2020	Eastern Campus, Renmin Hospital, Wuhan University, China	82 patients	78 umol/L
Ruan et al.	Intensive care medicine	March 2020	Jin Yin-tan Hospital and Tongji Hospital,	150 patients	91 in non-survivors 72 in survivors
Shi et al.	medRxiv	March 2020	Department of General Surgery, Renmin Hospital of Wuhan University,	101 patients	139.8 ± 22.83 umol/L

**Table 2.** Newcastle-Ottawa score of the included studies.

Study ID	Exposed cohort representative	Non exposed cohort selected from same source	Exposure ascertained	Outcome of study was not present at start of the study	Comparability	Adequate assessment	Follow up was long nough	Adequate follow-up	Quality score
Yang et al.	yes	Yes	yes	yes	1	yes	yes	yes	8
Cheng et al.	yes	Yes	yes	yes	1	yes	yes	yes	8
Zhou et al.	yes	Yes	yes	yes	1	yes	yes	yes	8
Zhang et al.	yes	No	yes	yes	0	yes	yes	yes	5
Ruan et al.	Yes	Yes	Yes	Yes	1	Yes	Yes	yes	8
Shi et al.	yes	No	yes	yes	0	yes	yes	no	5

**Figure 5.**
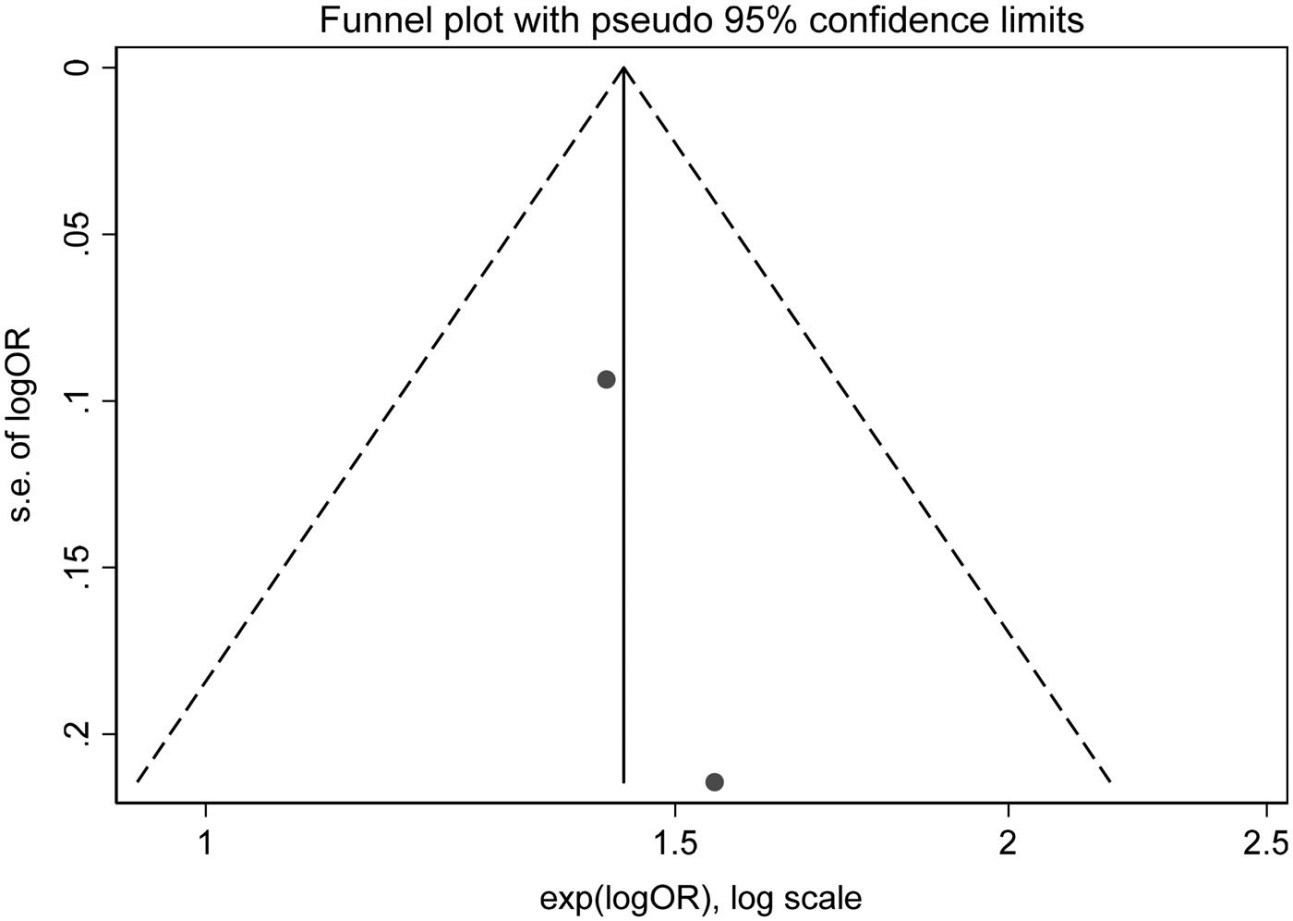
Funnel plot analysis after excluding Yang et al.

To decrease risk of heterogeneity, REM was repeated after excluding Yang et al (Figure 4). The association of severe AKI with mortality persisted (relative risk = 4.19, 95% CI 3.31 - 5.31). There was no evidence of heterogeneity with I-squared = 0%, *p* = .68. There was no evidence of publication bias when applying Eggers test (*p* < .05) or funnel plot analysis (Figure 5). Our meta-analysis supports that mortality is significantly higher in patients with severe AKI in patients with COVID-19. To date, the published incidence of AKI among patients with COVID-19 is highly variable. It has been reported to occur in up to 27% of patients with COVID-19 [12]. Our meta-analysis included three studies addressing mortality in COVID-19 patients with superimposed AKI. Cheng et al., included 701 COVID-19 confirmed cases. AKI stage III occurred among 14/701 (2%) of the patients and was associated with an increased risk of in-hospital mortality (hazard ratio = 9.81, 95% CI:5.46-17.65) [5]. Similarly, Yang et al. included 52 COVID-19 confirmed cases in their study and found that 8 out of 9 subjects who required RRT did not survive [13]. Mirroring Yang et al.’s results, Zhou et al., in a study that included 191 COVID-19-CC, 10 out of 10 subjects who required RRT did not survive [14]. In addition, the investigators reported that out of 33 confirmed COVID-19 cases who developed AKI, 32 patients did not survive [14]. The high mortality in COVID-19 patients and severe AKI, even with RRT, could be due to the kidney-lung crosstalk during COVID-19 infection and amplification of inflammation during AKI in a cohort with high incidence of acute respiratory distress syndrome [15]. Based on the available limited published data, severe AKI in patients with COVID-19 is an ominous clinical predictor and is associated with high mortality. Further studies are needed to understand the factors associated with worse outcomes among COVID-19 patients with AKI. Understanding those factors may guide care providers in making more informed dialysis eligibility decisions under conditions where resources are extremely limited.
